# Observations on Chorea Sancti Viti; with a New Theory of the Disease

**Published:** 1805-05-01

**Authors:** John Redman Coxe

**Affiliations:** Philadelphia


### Observations on Chorea Sancti Viti; *with a new Theory of the Disease: by* John Redman Coxe, M.D. *of Philadelphia.*

JOHN MAGNUS, aged forty, a taylor by profession, of a full habit and sanguine temperament, about June, 1801, began first to complain of irregular or convulsive motions of the head, mouth and hands. It appears that he has, for a long time previous to this, had irregular motions of his hands and head, which gave him no concern. About two years ago he laboured under syphilis. At his first application for assistance he was advised to abstain from liquor, to take regular exercise both morning and evening, and to avoid costiveness, and sitting up so late at night to work as usual. This advice he neglected; and in about two weeks fever supervened, with great costiveness, which was removed by Glauber's salts, and the occasional use of aloetic pills. His complaints augmented, and in about a fortnight more he began to complain of stupor and giddiness of the head. His pulse was frequent and quick, but not full. He was bled to fourteen ounces, and took senna, manna, &c. The giddiness still continued, and pain began again to recur in a few days, with almost constant coma and stupor. He was again bled, and purged with jalap and calomel. Stramonium, boiled in vinegar, was ordered, to bathe his head, and poultices of the same were applied to his feet every night, which appeared to relieve him much. The good effects gradually subsided, and its further use was omitted.

Previously to the last bleeding, in July, he complained much of weakness of the eyes, attended with a dull pain and slight inflammation. A wash of sugar of lead and white vitriol was prescribed, and six leeches to the temples, which all died soon after sucking. A soreness of the nose and throat rendering it probable that his syphilitic complaint had not been completely cured, he was put, for a short time, on the use of the opium and calomel pills.

As each bleeding appeared to weaken him, leaving him more stupid for upwards of a day, the lancet was employed more cautiously. All this time his appetite was remarkably good, and so continued till near the close of his illness. He still grew worse, and an involuntary discharge of urine took place. The stupor appearing considerable, and his pulse being fuller, with skin hot and bloated face, and irregular motions continuing, he was bled to eighteen ounces, after which he seemed much weaker the first day, but less stupid. He took nitrous powders for about a week, but seemed no better, except in an abatement of his fever. His pulse became weaker, but not less frequent. He now took valerian in ʒß. doses, and gradually increased it to ʒ i. thrice a day. This continued for a week, gave no relief, and it was then combined with snake-root eight or nine days longer, with little effect; during which period he was blistered three or four times. He now began to complain of increase of pain in his head and back, and repeated the antimonial powders.

The above account I received from the apothecary of the Dispensary, who, at this period, requested me to visit him. I recommended the continuance of the antimonial powders, with the addition of small doses of camphor, and applied cups to the temples, neck, and adjacent parts, and a large blister to the head. The preceding blisters were dressed with mercurial ointment, which was likewise employed in friction. His mouth became sore, and slight salivation ensued.

After pursuing the above plan for some time, it was discontinued, and columbo, rust of iron, and rhubarb, were given for some days with no beneficial effect. The blisters were repeated.

On the 14th of September the leading symptoms were increased; pulse quicker; skin hot. Ordered to lose fourteen ounces of blood in the afternoon. In the evening very feeble, with increased flow of urine. He appeared somewhat better the 15th. As he was costive, the laxative ingredients were ordered, and he was blistered behind the ears. In the evening he seemed better, with moist skin, weak pulse, and continued increase of urine. The nitrous powders were continued. He was slightly delirious on the 18th; pulse fuller and irregular. The powders were continued. On the 19th, pulse regular but feeble; continue powders, and give a tonic medicine of bark, Port wine, and laudanum. In consequence of the increase of febrile symptoms, he soon after lost eighteen ounces of blood. The medicines were continued till the 28th of November. From the recommendation of the Medical Repository, Hex. I. vol. v. p. 95, as extracted from Hufeland's Journal, I began the use of phosphorus in the following manner, after he had for a week omitted all his medicines.

October

October 4th. ℞. Phosphori grs. ij. laudani gtt. xl. white sugar ʒij. gum arab. ℈i. Rub them well together in a glass mortar, and add tr. of cinnamon ℥i. water, &c. Make ℥. viij. Of this he was directed to take a table-spoonful every hour. He began it in the evening.

5th. Symptoms continue; no apparent effect from the medicine.

6th. Pulse generally from 80 to 100; symptoms as yesterday. Bowels open; appetite good; has taken about one grain of phosphorus in the last twenty-four hours; repeat it to two grains in twenty-four hours.

7th. Convulsive motions apparently less; seems languid; pulse only 56; eyes look natural.

8th. Pulse 60; appetite, if any thing, greater than usual; symptoms as above: continue the phosphorus to two and a half grains in twenty-four hours.

9th. Low and languid through the day; no medicine taken of the last increased quantity. He fainted this morning; and walked across the room with difficulty, supported. Towards night-fall was attacked with a violent vomiting, continuing a considerable time; for which his wife gave him sixteen drops of laudanum. This was vomited, but it then ceased, and he slept a little, with much rambling. No stool since yesterday morning. Towards midnight vomiting returned, with considerable dyspnœa, and much apparent debility.

Ordered, early on the morning of the 10th, a mixture of bark, wine and laudanum. At nine A. M. I found his breathing stertorous; no stool; vomiting stopped, but appearing to suffer in his stomach, which, as we understood, pained him. Pulse tremulous, and scarce perceptible at the wrist, but more full and slow above the elbow; feet cold to the ancles; the arms also are cold, owing, in part, probably, to his frequently exposing them from beneath the bed-clothes. Considering him now, as I should have done before, had I comprehended the theory of chorea, as labouring under a depressed state of the system, I tried to bleed him, but could not obtain more than two or three tea-spoonfuls of blood, which appeared to me to be in a dissolved state. I therefore ordered him an emetic of twenty grains of white vitriol, and four of tartar emetic, dissolved in half a pint of water, to take a table-spoonful every ten minutes, till it should operate, in order to relieve the stomach, if any thing he had taken had affected him. One half was ineffectually taken, when I ordered a double dose of castor-oil without effect. At noon he continued the same. Pulse imperceptible at the wrist, and no vein visible to try venesection. Ordered senna and manna a. ℥ i. rhubarb ʒß. Infuse in a pint of boiling water, and give a large tea-cupful every hour, as also a powerful purgative enema every hour. Cataplasms to the feet, and bleed if possible. Six o'clock P. M. Medicines all taken, and six enemas, without effect. Some wind was discharged. Ordered calomel ℈i. gamboge grs. x. in a dose of Castor oil, immediately, and repeat the oil every hour if necessary. Boil up the former ingredients with a pint of water, and add Glauber's salts ℥ j. and use for the glyster, instead of common water, with two table-spoonfuls each of salt and molasses. Apply a blister to the stomach; pulse imperceptible; no pain of the head apparently; pupils natural; convulsions diminished; breathing the same, and very slow; much rambling, with slight intervals of reason. About nine P. M. he died.

DISSECTION.

October 11th, eleven o'clock.—The body, on inspection, had no symptoms of emaciation; and accordingly, on cutting the teguments, we found the fat on the abdomen not less than one inch thick, and in some parts considerably more. The omentum and mesentery were uncommonly loaded with the adipose matter. Stomach and intestines much distended with flatus. Stomach, internally, slightly inflamed, which I was rather disposed to consider as the effect of the gastric fluid after death, as no disposition to vomit had existed for more than twenty-four hours preceding his death, which would scarcely have been the case had the inflammation existed before that event. Kidnies, spleen, pancreas, and gall-bladder, perfectly sound. The liver was enlarged, and cut as if slightly schirrous; and its external appearance much resembled Castile soap, with small streaks and obscure white. The urinary bladder was healthy, though contracted, owing to his long incontinence of urine, originating, doubtless, in the affection of the brain, hereafter to be noticed.

The thoracic viscera were sound, the lungs adhering partially to the surrounding pleura. The fat in great abundance around the basis of the heart, and no unusual proportion of fluid in the pericardium.

On separating the scalp the vessels bled very much, and much blood oozed from the small vessels passing from the pericranium to the bones of the head. After raising the cranium, the vessels were turgid with blood, the small ones bleeding freely when ruptured from the bone. Several veral portions of coagulable lymph were found in the longitudinal sinus, and the extremities of the large vessels emptying into it. Great inflammation was apparent, the whole length of the sinus, on its edges, where it touches the hemispheres of the brain, extending itself on the surface of the pia mater, and about the blood-vessels. Firm adhesions existed between the dura and pia mater at this part, by considerable portions of perfectly organic coagulable lynph, most evident about the coronal suture.

On cutting into the substance of the brain, the blood oozed from every extremity of the divided vessels; and, from the number, I concluded that many now conveyed red blood, which, in health, admitted a pellucid lymph alone. On cutting cautiously down, I completely separated it, for some length, from the thin transparent pia mater, which lines the ventricles, and which was here greatly distended by at least twelve ounces of a very transparent fluid.

The various communications between the ventricles were here most beautifully exhibited, especially the foramen ovale uniting the two lateral ventricles, which was here large enough to admit, with ease, the extremity of the little finger. The blood-vessels of the cerebellum were equally turgid. The optic nerves appeared sound. The dissection occupied above two hours.

### Observations.

As I did not see this patient for nearly three months from his attack, I can say nothing of his case, except from the report of the person attending, which is fully detailed. I had little suspicion at first of water in the brain, as he complained of very little pain in it at any period after I saw him; neither was it accompanied with strabismus, or dilated pupil. He experienced none of those changes in the pulse peculiar to hydrocephalus, its uniform state being from 70 or 80 to 100, except after the use of the phosphorus, when it fell to 56. At the time, I supposed it owing to the medicine, and probably it was so, by exciting an increased determination of blood to the brain, and thus inducing apoplexy, or acute hydrocephalus. From the time I saw him he was affected with fatuity, and towards the close of the disease he seemed to have indistinct vision. Till within about ten days of his death, he could distinguish the hour of th day in a watch at least six feet dsstant from him. He saw double occasionally about two days before his death. His appetite was always good, and even voracious, and I never could restrain it till within two days of his exit. He always asserted his head was well, and complained only of great debility. His pulse never beat very hard (or rather it was depressed), and the blood was sizy and cup-shaped. He lost altogether about an hundred ounces of blood, which gave relief, though drawn in quantities of eighteen and twenty ounces at a time. Blisters to the head, neck, ears, and wrists, and kept running for a considerable time, gave no ease; and though under the influence of mercury for nearly six weeks, during three of which he spat considerably, with sore gums and loose teeth, yet it gave no respite. Nitrous powders every two hours, so as to induce nausea and vomiting, did not affect his complaint, either for better or worse. His bowels were mostly laxative. Digestion went on well, as the immense quantity of fat evinced; and but a small portion of fœcal matter was found in the alimentary canal.

The water must have been accumulated a considerable time, as evinced by the quantity, and the very enlarged state of the foramen ovale. Its sudden effussion must have produced apoplexy; but the slow progress of the effusion permitted the brain to accommodate itself to the pressure, by the action of its absorbents upon the substance of the brain itself, and serves to evince the power of the system to resist a morbid impression, when slowly applied.

The water in the brain was not, I believe, the immediate cause of death. Some sudden effusion or congestion was the source of the fatal issue, by producing apoplexy. The turgid state of the blood-vessels would seem to favour this opinion. Whether the phosphorus induced this I cannot say. He took about four grains in all. I have supposed it was the source of the event which took place, and have never since used it.

Had I viewed the disease in the light I now do, I should have depended on copious evacuations from the bowels and blood-vessels, with blisters; but I confess I was in a great measure in the dark as to the cause of his illness.

The case which I have thus faithfully detailed, led me to think more seriously on the subject of the chorea than I had before done, and convinced me it ought not to be viewed in the light of an idiopathic disease. A case, hereafter detailed, which very lately came under my care, has added strength to the opinion I entertain; and a very interesting one, related by Dr. Paterson, in his "Letters concerning concerning the internal Dropsy of the Brain," has served but to confirm it in my mind.

The Chorea Sancti Viti is, as I hope to prove, merely a symptomatic affection of a disease which, though long known, has not, I think, been thoroughly understood till of late years: I mean Hydrocephalus Internus. That the chorea has never been properly elucidated, is, I think, apparent, from the numerous opinions which have been entertained respecting it. Some have considered it as of the epileptic kind; some of the paralytic; and others of both. It has been viewed as a disease of the muscles, considered as a branch of the nervous system. It has been regarded as similar to the disease produced by the bite of a tarantula, and it has also been ascribed to witchcraft. By all it has been passed over so cursorily that little is to be gained, even of the facts connected with its history. Even Sydenham, who has, perhaps, given us the best description of the disease, scarcely adverts to any thing but its curious gesticulations.

However numerous may be the *remote* causes of a disease, the *proximate* must necessarily be uniform; and of the proximate cause of chorea I cannot trace the slightest suspicion in authors. Dr. Patterson, in his treatise above alluded to, has come so nigh as to place the cause for the effect, as he considers chorea the original disease, and attempts to explain the manner in which convulsive affections produce watery effusions in the brain; a fact he affirms to be sufficiently attested by dissection. I shall hereafter give a short view of the case he details, as far as it may serve to substantiate my opinion, and proceed in my attempt to develope the pathology of the disease, by proving that it arises *solely* as a symptom of the *chronic* state of hydrocephalus internus.

In general, we know hydrocephalus to be a disease rapid in its termination. It certainly, however, exists frequently as a chronic affection, as numerous dissections of Bonetus, Morgagni, and others, testify. When it comes on thus slowly, it permits the brain to accommodate itself to the pressure of the fluid in the ventricles, doubtless by a portion of its substance being taken up by the absorbents, without producing its direful symptoms in their full extent; but inducing various grades of apoplexy, epilepsy, palsy, and occasionally of this curious affection, chorea.

Hydrocephalus generally attacks children; so does chorea; and both occasionally occur in adults. The symptom of strabismus does not invariably take place in hydrocephalus; and though mostly absent, yet it sometimes occurs in chorea. The cause in both must be the same.

Hydrocephalus sometimes follows inflammatory fever, where the head has been much affected; so does chorea; and both sometimes occur from blows on the head.

A too rapid growth of the body in early life may, by the determination to the brain, excite hydrocephalus; and chorea sometimes appears to depend on the same remote cause for its production.

A sudden check to accustomed secretions, such as discharges from the ears, &c. sometimes appears the remote cause of both diseases.

Habit, doubtless, may keep up this curious affection long after the original cause is removed. Hence also frights are said to produce its recurrence, of which De Haen furnishes us with an example, after a lapse of more than two years. We are told, however, by Sydenham, that those once affected with it are apt to have it recur. I should, therefore, rather ascribe it to a renewed affusion, by a sudden determination to a weak part. Chorea is not, in general, noticed till some time has elapsed. It is, however, probable that, could it be attended to from its earliest attack, we should observe many obscure symptoms of water in the brain. Aphonia, idiocy, &c. are not uncommon from hydrocephalus; and they also occur in the higher grades of chorea. The muscles of the face are affected in both diseases, producing spasmus, cynicus, risus sardonicus, &c.

Girls are said to be more frequently affected with chorea than boys. Is this really the case, or is it merely accidental? And how is the fact as it respects hydrocephalus?

Some have supposed this disease to depend on worms, and hydrocephalus has been also ascribed to them. The symptoms of worms are so numerous, according to many authors, that, like some other complaints, they are seen lurking in every disease. For my part, I confess I think them a very harmless and highly persecuted part of the creation. We find them uninjurious in many healthy children. Few, perhaps, pass the stages of infancy and childhood without them. If they exist in great abundance, I can suppose them very prejudicial; and perhaps they are likewise detrimental when disturbed themselves by some febrile commotion in the system, which they may then augment, but certainly did not produce. For how is it possible that such violent symptoms should suddenly arise from the presence of one, two, or even a dozen of of these creatures; and yet, during the progress of their growth, nothing of the sort take place? Should not the symptoms exist proportioned to their increase? The remedies which have been effectual in removing some diseases, having produced the evacuation of any worms in the alimentary canal, have been falsely supposed to have cured by their removal. How rarely do we find many of these creatures evacuated by the various anthelmintics employed for their removal; and how often do we find the symptoms ascribed to worms vanish, after the operation of the medicine given, without the evacuation of a single one! I shall not digress further here on the almost universal quackery in families on this subject, though much might be said. I will only add, that I fear many children are carried off by dropsy in the brain, whilst the anxious parents are trying every remedy, public and private, to remove worms, which exist only in their own brains.

If hydrocephalus is the cause of chorea, it may be asked, as the former is not an unfrequent disease, why the latter does not more often occur? I cannot pretend to resolve the question; but may ask, why the apoplexy, epilepsy, palsy, &c. are not always produced? We know too little of the nervous system to reason accurately upon it; yet I feel persuaded of the truth of the above principles, although I cannot, perhaps, agree conclusively upon them.

It may be asked, also, why chorea is not, in general, a fatal disease, if it arises from dropsy of the brain? This, I think, can only be explained upon the principles of habit; the slow deposition of the fluid in the ventricles allowing the brain to accommodate itself to the burden. When death occurs in chorea, it seems to me probable that it arises from some sudden increased determination to the vessels of the brain, producing either a kind of acute hydrocephalus, or an engorgement of the vessels themselves; in either case inducing apoplexy. It is to be observed that, although the brain was habituated to a certain degree of distention, yet any *sudden* addition to that must act precisely as if such habit had never been acquired.

Man is not the only animal subject to chorea, as may be readily supposed. I find, however, no notice taken of its occurrence in any other animal by those authors I have perused. I have been lately informed by a lady, that she knew a whole litter of pigs suffer under the disease till they were upwards of three months old. And I have have now in my possession a dog, of the coach-house breed, whose left fore leg and side, together with the left side of his face, have been greatly affected with it ever since an attack of the distemper in the last autumn. He was much benefited by two or three active mercurial purgatives; and at the time of my writing this, he is almost well.

This leads me to say, that I think the analogy of diseases in man and beasts are not sufficiently attended to. This disease (the distemper) has many symptoms, as well as the staggers or convulsions of horses, in common with hydrocephalus. In my opinion (without the certainty of demonstration by dissection) they are all the same disease. Taplin, who has written upon the various diseases incident to that noble quadruped the horse, has not thought it beneath his notice to treat of the distemper of dogs, and has, doubtless, rendered the disease less formidable by his judicious treatment. He considers the complaint, however, as affecting particularly the throat, stomach, and intestines. I regret, however, his neglect to ascertain this important point by dissection, especially as the disease, before his observations on it, was generally viewed as seated in the head. I have never yet had an opportunity of verifying or rejecting my opinion by dissection since I first entertained it. There are, however, several circumstances mentioned by him in favour of my ideas.

The disease appears at a period of life in some measure analogous to that period in which hydrocephalus most usually occurs in the human subject; but the cause he mentions would be likely to occur at any period, and even to be frequently repeated; whereas the animal seldom suffers from a second attack. I cannot easily explain, upon either opinion, why it more frequently, and with greater violence, attacks the females than the males. The debility, in the latter stage of the complaint, of the hinder parts, amounting nearly to paralysis, and the frequent twitchings and convulsive spasms, appear to me more nearly connected with effusion in the ventricles of the brain than with the violent obstruction of the stomach or some part of the intestines. The obstruction he speaks of, I apprehend, may be viewed more as a remote than a proximate cause; whilst his powerful cathartics, combined with tartar emetic, &c. appear to favour the absorption of fluid in the ventricles of the brain. The rapidity of the cure will not be considered extraordinary, when we we recollect the speedy discussion of distant tumours, sometimes by the operation of an emetic: and the smallest portion of fluid re-absorbed would be sufficient to diminish the immediate danger. After all, this disease can be understood with certainty only by dissection, which I ardently anticipate: viewing it, however, as distantly related to the subject before us, I thought the digression might be serviceable to a very useful class of animals.

In confirmation of the chronic state of hydrocephalus being the probable cause of chorea, I shall refer to Willis, in his Treatise on Convulsive Diseases, where are detailed several interesting cases of convulsions of the muscles, &c. for several years; dissection after death shewing the brain to be filled with water, which had doubtless produced and continued the disease.

The motions in chorea cease during sleep, with, perhaps, few exceptions. In the last case I saw, the eyelids continued partially open, and the eyes rolled considerably.

The will, I doubt not, has some effect on the disease, as the motion is occasionally restrained by fear. Cullen talks of its having been epidemic in some part of Scotland. If so, I think it must have been partly owing to an imitative propensity, as in the epilepsy, related by Kaauw Boerhaave.

I cannot explain why the disease should be sometimes partial, sometimes universal; nor why, according to De Haen, of ten cases he saw, nine should be of the left side. Partial pressure, may, perhaps, explain the former: the latter I should presume to be merely accidental.

This disease differs from tetanus (independently of its cause), in as much as the flexors and extensors of the limb, or antagonist muscles, are in a constant and rapid succession, though unequal motion; whilst in tetanus one or other set generally predominates, or else both act with equal force. On this account it is, that it is usually not a painful disease, as no one muscle contacts so long as to cause pain by so permanent a state. And from this rapid *unequal* motion of each antagonist it probably is that such strange contortions are made, preventing a cup, &c. from being brought in a direct line to the mouth.

Hysteria is sometimes combined with chorea, as in the case hereafter mentioned, as is also catalepsy.

Are not the convulsions of acute hydrocephalus analogous to chorea in the chronic?

Sauvages, in his Nosologia Methodica, in speaking of this disease under the name of Scelotyrbe, or Dance de St. Guy,

St. Guy, mentions one species of it as intermitting. (Vol I. p. 592.)

In the cure of this disease, De Haen speaks highly of electricity, though more, I think, than it deserves. Rotheram, in his Notes on Cullen's First Lines, mentions a case in which it was serviceable. Much of its utility probably depends on the period of its application.

Camphor has been employed in this disease. A case is given in one of Duncan's Medical Commentaries, in which it was employed for three months. The tonic plan has failed at first, probably from being used too soon, or when depletion was necessary. Though the credit of the cure, in this case, is given to the camphor, I must confess I think it as likely to have been effected by the gradual tone acquired by the system from other sources.

The reason why the remedies in general use (tonics) are, for the most part, successful, is, I imagine, that we are called in at that period when absorption is most readily excited by the general tone they give the system. In the early stages it often requires bleeding and purging, as was Sydenham's method; and which, with little addition, is the best mode of removing hydrocephalus. Sydenham has laid it down as an universal measure to bleed; but this must depend on the state of the system as indicated by the pulse. If it is active, bleeding is certainly proper; after which I shall pursue a mercurial course, and follow it up with tonics, as bark, columbo, steel, elixir of vitriol, port wine, and the cold bath.

I shall now give a brief statement of the case related by Dr. Patterson, at page 58 and seq. of his treatise.—In this case, a girl of nine years of age, a frequent and tormenting head-ach of some years continuance, accompanied with severe stomach sickness, had preceded the Doctor's first visit on the 3d of April, 1790, for chorea, which had been discovered a few weeks preceding, especially of the *left* side. The *head was now free from pain;* pulse slow and small; eyes *regular* and *sound;* bowels costive; and coldness and impaired use of the *left* arm. On the 22d she had a slight head-ach, for the fourth time since her illness. Under the use of tonics of various kinds, and electricity, the chorea appeared to mend during the month of May. But on the 13th of June she again complained of the violent head-ach and sick stomach; for which, on the fourth day of its attack, a vomit was administered, with little benefit; some involuntary motion of the *right* hand, and vision less distinct, &c. On the 18th her head was was so bad that she took a second emetic, with some relief; but she was as bad the following morning, continuing to the 22d. Her eyes were proper. The *convulsions had gone,* and she had *regained the perfect use of her hands.* A blister relieved the head-ach; but a paralytic stroke followed in the course of the day; the mouth being distorted, and the *right* arm having lost its use. On the 25th, *Strabismus* towards the left side; *dilated* pupils; insensible to light; *convulsively* affected in the *left* side; *torpid* in the *right.* On the 26th the left arm was more convulsed, the right remaining torpid; at midnight general convulsions recurring, of various degrees. At two o'clock she died.

On being examined, the blood-vessels of the dura mater were turgid and reddish, exhibiting appearances of strong preceding inflammation. A kind of serous diffusion existed over the circumvolutions of the lobes; the left lateral portion of the plexus choroides was inflamed; and two ounces of water were found in the left ventricle, and half that quantity in the right.

In this interesting case, which is fully detailed by Dr. Patterson, I feel no doubt of the long continued and violent head-achs being owing to the fluid in the ventricles of the brain. The alternation of the head-achs with chorea, show strongly the connection of the two diseases; whilst the paralysis of the *right* arm, and the convulsions of the *left,* show the connection of chorea and palsy. The operation of the emetics probably hastened her dissolution, by determining too forcibly the circulation of the brain, and hence exciting an acute state of hydrocephalus.

It appears to me that Dr. Patterson has greatly erred in supposing chorea the primary disease; of which each must judge for himself.—I shall now beg leave to relate a case of chorea which has lately come under my care, and then dismiss the subject.

December 15th, 1803.—Rosetta, aged nine years, a mulatto child, hitherto perfectly healthy, but very mischievous, for which she had repeatedly suffered from blows, &c. When an infant, she lost, by an accident, all the toes of the right foot except the great toe. About six weeks ago she began to be affected with those convulsive motions of the right arm and foot, which have since so much increased. They were at first thought to be owing to mischief, as she let every thing fall she took in her right hand, which was, till within a few days past, the part principally principally affected. Her face and tongue at length partook of the complaint, and she now talks but little; though at times she screams out greatly, yet without complaining of any pain. Her tongue is white, and pulse active, though not very quick. The eyes are not affected, and she sleeps sound, without any of those convulsions which affect her during the day. I ordered her ten grains of calomel at bed time, and a scruple of rhubarb in the morning—to lose eight ounces of blood, and to be put on low diet. The medicine operated well, and blood was natural. Her left side became now greatly affected. Pulse is moderate. I therefore prescribed, on the 17th, fifteen grains of bark, and ten of rust of iron, three times a day, with a grain of opium night and morning. The symptoms still increasing, I omitted the bark and steel, and on the 21st put her on a mercurial course by friction, with antimonial powders (composed of two grains of nitre, two of calomel, and one fourth of a grain of tartar emetic) every two hours. She complains of pain on the left side of her head since yesterday, for which I blistered her behind the ears. I learnt to-day that she had been frequently employed by a gentleman, whom I had had under my care in the same house, to rub in mercurial ointment upon his legs, which she did with her bare hands, and continually was dabbling in cold water. Quere, its effects upon her? She has hitherto been under little controul; yet I found I could readily intimidate her, which now became absolutely necessary, as whenever she was in the least displeased, she screamed and roared so as to alarm the neighbourhood. On the 26th her mouth became very sore, from the medicine, which was consequently suspended: but as she had frequent hysteric paroxysms of laughing and crying, I used assafœtida in small doses. The great source of uneasiness to her, and which evidently made her worse, is an impression, which she and the neighbours gave way to, that she is bewitched by an old woman who lives near her, and is very ill, and whom she thinks she sees every night. By threatening to apply a blister if she continued to see the old woman, under the plea of its being a strong proof of her disease being worse, I in some measure destroyed her foolish apprehensions; but the death of the old woman, about the beginning of the year, did more for me than all my threats; for she never saw her afterwards. Little was done for her at this time, except giving gentle doses of physic, as her teeth were very loose, and the spitting continued. On the 2d of January, 1804, I began the use use of tonics; columbo-root and steel a. ʒij. made into twelve doses; one three times a day; and on the 7th increased the steel to ʒiij. all with evident good effect. I have omitted to mention the motion of her eyes during sleep, although the convulsions of her head, &c. were totally suspended. She was now able to walk much more steadily, and mended daily. On the 11th, in addition to the powders, I ordered the shower-bath, with the happiest results. At this time I was struck, in her walking, with a kind of cataleptic affection — a sudden and involuntary stopping for nearly half a minute at a time, when she would again resume her walk. By the 23d she was nearly well; scarce a vestige of the disease remained; and her fatuity and idiotic appearance seemed quite gone; a few slight twitches of her fingers alone remaining, as if from habit merely. The cold bath for about a week longer completed her recovery.

*March* 20, 1804.

